# Prediction of RNA-protein sequence and structure binding preferences using deep convolutional and recurrent neural networks

**DOI:** 10.1186/s12864-018-4889-1

**Published:** 2018-07-03

**Authors:** Xiaoyong Pan, Peter Rijnbeek, Junchi Yan, Hong-Bin Shen

**Affiliations:** 1 Department of Medical Informatics, Erasmus Medical Center, Rotterdam, The Netherlands; 2 Institute of Software Engineering, East China Normal University, Shanghai, China; 3 Institute of Image Processing and Pattern Recognition, Shanghai Jiao Tong University, and Key Laboratory of System Control and Information Processing, Ministry of Education of China, Shanghai, China

**Keywords:** RNA-binding protein, Sequence motifs, Structure motifs, Convolutional neural network, Bidirectional long short term memory network

## Abstract

**Background:**

RNA regulation is significantly dependent on its binding protein partner, known as the RNA-binding proteins (RBPs). Unfortunately, the binding preferences for most RBPs are still not well characterized. Interdependencies between sequence and secondary structure specificities is challenging for both predicting RBP binding sites and accurate sequence and structure motifs detection.

**Results:**

In this study, we propose a deep learning-based method, iDeepS, to simultaneously identify the binding sequence and structure motifs from RNA sequences using convolutional neural networks (CNNs) and a bidirectional long short term memory network (BLSTM). We first perform one-hot encoding for both the sequence and predicted secondary structure, to enable subsequent convolution operations. To reveal the hidden binding knowledge from the observed sequences, the CNNs are applied to learn the abstract features. Considering the close relationship between sequence and predicted structures, we use the BLSTM to capture possible long range dependencies between binding sequence and structure motifs identified by the CNNs. Finally, the learned weighted representations are fed into a classification layer to predict the RBP binding sites. We evaluated iDeepS on verified RBP binding sites derived from large-scale representative CLIP-seq datasets. The results demonstrate that iDeepS can reliably predict the RBP binding sites on RNAs, and outperforms the state-of-the-art methods. An important advantage compared to other methods is that iDeepS can automatically extract both binding sequence and structure motifs, which will improve our understanding of the mechanisms of binding specificities of RBPs.

**Conclusion:**

Our study shows that the iDeepS method identifies the sequence and structure motifs to accurately predict RBP binding sites. iDeepS is available at https://github.com/xypan1232/iDeepS.

**Electronic supplementary material:**

The online version of this article (10.1186/s12864-018-4889-1) contains supplementary material, which is available to authorized users.

## Background

RNA-binding proteins (RBPs) are highly involved in various regulatory processes, e.g. gene splicing and localization, and provide important functional information for patient care [1]. Finding the binding sites of the RBPs is therefore an important research goal. Studies have shown that RBPs bind to RNA molecules by recognizing both sequences (sequence motifs) and secondary structure contexts (structure motifs) [2–4]. For example, the amyotrophic lateral sclerosis associated protein FET binds to its RNA target within hairpin and loops structure [5]. RBPs specifically recognize loop and stem regions of miRNA precursors to regulate miRNA expression level [6].

The current limited set of known RBPs have been found using time-intensive and expensive high-throughput technologies such as RIP-seq and CLIP-seq [7]. Therefore, recent research has focused on the development of several fast and low-cost discovery tools for sequence-motifs and structure-motifs as shown in Table 1. Some tools only search for sequence motifs. The widely used MEME model fits a mixture model using expectation maximization to discover multiple sequence motifs [8]. MatrixREDUCE infers the sequence-specific binding motifs for transcription factors [9]. Other tools also take secondary structure into consideration to predict the binding site. MEMERIS searches for RNA motifs enriched in regions with high structural accessibility [2]. BEAM identifies represented structure motifs from sets of unaligned RNAs by considering the evolutionary information [10]. Li et al., integrate the accessibility of RNA regions around the RBP interaction sites to identify accessible sequence motifs [3]. CapR models the joint distribution of residue positions and secondary structures to identify the binding sites under different structure context [11]. RNAcontext trains machine learning models using sequence and accessibility information to infer sequence and structure motifs [12]. GraphProt [13] integrates the RNA sequence and secondary structural contexts using a graph kernel model to investigate the RBP binding preferences, and it represents input sequences using over 30,000 dimensional graph features. Recently, the iONMF [14] integrates kmer sequence, secondary structure, CLIP co-binding, Gene Ontology (GO) information and region type using orthogonal matrix factorization to predict binding sites.

**Table 1:** Computational methods for RBP binding preference prediction

Method	Sequence motif	Structure motif	Model	Code	Reference
MEMERIS	Yes	No	Maximum likelihood estimation	http://www.bioinf.uni-freiburg.de/~hiller/MEMERIS/	[2]
BEAM	No	Yes	Simulated annealing	http://beam.uniroma2.it/	[10]
CapR	No	Yes	Turner energy model	https://sites.google.com/site/fukunagatsu/software/capr	[11]
Li et al.	Yes	Yes	Iterative refinement	-	[3]
GraphProt	Yes	Yes	Graph encoding	http://www.bioinf.uni-freiburg.de/Software/GraphProt/	[13]
DeepBind	Yes	No	CNNs	http://tools.genes.toronto.edu/deepbind/	[19]
DeeperBind	Yes	No	CNNs and LSTMs	https://github.com/hassanzadeh/DeeperBind	[23]
RNAcontext	Yes	Yes	probabilistic models	http://www.cs.toronto.edu/~hilal/rnacontext/	[12]
Zeng et al.	Yes	No	CNNs	http://cnn.csail.mit.edu	[24]
iDeep	Yes	No	DBNs and CNNs	https://github.com/xypan1232/iDeep	[28]
iDeepV	No	No	CNNs	https://github.com/xypan1232/iDeepV	[22]
iDeepE	Yes	No	CNNs	https://github.com/xypan1232/iDeepE	[29]
iONMF	Yes	No	matrix factorization	https://github.com/mstrazar/iONMF	[14]
Deepnet-rbp	Yes	Yes	DBNs	https://github.com/thucombio/deepnet-rbp	[21]
DanQ	Yes	No	CNNs and LSTMs	http://github.com/uci-cbcl/DanQ	[27]

The methods discussed above require domain knowledge to hand-design the input features. For example, we need to first extract discriminate features, e.g. region type and clip-cobinding [14], with domain-specific knowledge for predicting RBP binding sites. To remove the need for prior knowledge, fully data-driven approaches, such as deep learning [15, 16], are being developed. Deep learning has proved to be very successful in many research areas, e.g. image recognitions [17] and information retrieval [18]. Promising performances were also demonstrated on predicting RNA-protein interactions and binding sites [19–22] (Table 1). For instance, DeepBind applies CNNs to automatically capture the binding sequence motifs [19]. DeeperBind added another long short-term memory network (LSTM) layer to learn dependencies between sequence features to enhance protein-DNA prediction [23]. Zeng et al. provides a flexible framework for selecting CNN architectures to predict DNA-protein binding [24]. Deepnet-rbp incorporates structure features using deep belief networks (DBNs). It includes the RNA structure information, obtained from another tool, as a count vector of kmers [21]. A disadvantage of Deepnet-rbp is that it requires complicated steps to estimate the binding preference [21]. Apart from CNN-based methods, LSTM is also widely used in predicting subcellular localization of proteins, precursor miRNAs and DNA-protein interaction [25–27]. For example, DanQ applies LSTMs to capture long-term dependencies between the motifs identified by CNNs [27].

Our previous iDeep model predicts the RBP binding sites on RNAs and sequence motifs using the hybrid CNNs and DBNs by integrating multiple sources of hand-designed representations, including region type and clip-cobinding [28]. iDeepE trains local and global CNNs to infer sequence binding motifs [29]. However, similar to DeepBind [19], it can discover only the sequence binding preferences. In this study, we propose and evaluate an improved version, called iDeepS, which consists of CNNs and a bidirectional LSTM. The iDeepS method identifies the sequence and structure binding motifs simultaneously. To the best of our knowledge, iDeepS is the first method to fully automatically capture both the sequence and structure binding motifs using CNNs.

## Results

**Fig. 1:**
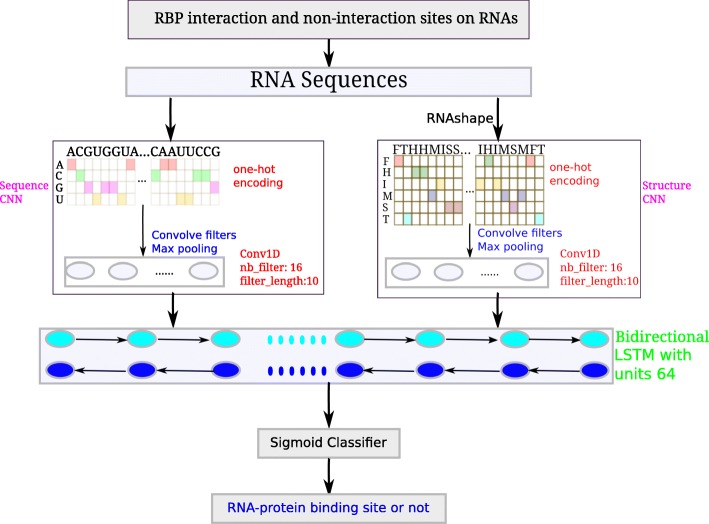
The flowchart of proposed iDeepS. For each experiment, iDeepS integrates two CNNs (one is for sequences, the other is for structures predicted by RNAshape from sequences) to predict RBP interaction sites and identify binding sequence and structure motifs, followed by the bidirectional LSTM, which learns the long range dependencies between learned sequence and structure motifs. Finally, the outputs from bidirectional LSTM are fed into a sigmoid classifier to predict the probability of being RBP binding sites

In this study, we evaluate iDeepS on large-scale RBP binding sites derived from CLIP-seq [30]. Figure 1 shows the flowchart of iDeepS for predicting RBP binding sites. The details of the network architecture are shown in Additional file 1: Figure S1. We evaluate the performance of iDeepS for predicting binding sites on RNAs and compare it with the state-of-the-art methods. Furthermore, we identify the binding sequence and structure motifs using CNNs integrated in iDeepS.

### Performance of iDeepS

The performance of iDeepS is compared with both sequence-based and structure-based methods as described below.

First, we compare it with the sequence-based DeepBind and Oli across the 31 experiments. iDeepS results in an average AUC of 0.86, which is a little better than 0.85 of DeepBind, and similar to AUC 0.86 of DeeperBind. The performance of Oli [31] is much lower than iDeepS, with an average AUC of 0.77 across the 31 experiments. For some proteins, Oli’s performance is close to random guessing, e.g. protein Ago2-MNase with AUC 0.512. As showed in Table 2, iDeepS outperforms DeepBind on 25 of 31 experiments, DeeperBind on 19 experiments, and Oli on all experiments. It is interesting to note that the three methods have large performance differences across individual experiments. For iDeepS, the AUCs ranges from 0.59 for protein Ago2-MNASE to 0.98 for protein HNRNPC. For Ago2 protein, iDeepS cannot yield high performance. The reason is that Ago2 binding specificity is primarily mediated by miRNAs [32], the expressed miRNAs have a high influence on Ago2-RNA interactions, which results in more variable binding motifs than RBPs that bind to RNAs directly. In addition, we compare iDeepS with DBN-based DBN-kmer that uses kmer features and a DBN to predict RBP binding sites. DBN-kmer yields the mean AUC of 0.77 (Additional file 2: Figure S2), which is much worse than CNN-based DeepBind and iDeepS.

**Table 2:** The AUC performance comparison between iDeepS and other methods on 31 experiments

Protein	iDeepS	DeepBind	DeeperBind	Oli	GraphProt
1 Ago/EIF	**0.773**	0.713	0.740	0.610	0.691
2 Ago2-MNase	0.591	0.595	**0.606**	0.512	0.595
3 Ago2-1	**0.865**	0.849	0.857	0.803	0.817
4 Ago2-2	**0.868**	0.830	0.868	0.800	0.823
5 Ago2	**0.634**	0.628	0.630	0.534	0.633
6 eIF4AIII-1	**0.950**	0.938	**0.950**	0.919	0.918
7 eIF4AIII-2	0.953	0.950	**0.954**	0.929	0.931
8 ELAVL1-1	**0.932**	0.924	0.930	0.889	0.915
9 ELAVL1-MNase	0.600	0.613	**0.614**	0.491	0.591
10 ELAVL1A	**0.893**	0.886	**0.893**	0.843	0.867
11 ELAVL1-2	**0.919**	0.914	**0.919**	0.875	0.895
12 ESWR1	**0.917**	0.912	0.915	0.808	0.840
13 FUS	0.934	**0.942**	0.939	0.846	0.860
14 Mut FUS	**0.958**	0.953	0.957	0.822	0.853
15 IGFBP1-3	**0.717**	0.702	0.713	0.569	0.697
16 hnRNPC-1	**0.960**	0.957	0.959	0.885	0.930
17 hnRNPC-2	0.975	0.973	**0.976**	0.941	0.953
18 hnRNPL-1	0.756	**0.771**	0.746	0.392	0.698
19 hnRNPL-2	0.747	**0.769**	0.746	0.474	0.708
20 hnRNPL-like	0.708	**0.711**	0.679	0.562	0.650
21 MOV10	**0.813**	0.804	0.812	0.783	0.803
22 Nsun2	**0.835**	0.803	0.801	0.754	0.779
23 PUM2	**0.962**	0.950	0.955	0.939	0.914
24 QKI	**0.966**	0.962	0.961	0.924	0.932
25 SRSF1	**0.887**	0.874	0.875	0.839	0.838
26 TAF15	**0.964**	0.956	0.963	0.804	0.850
27 TDP-43	**0.930**	0.926	**0.930**	0.883	0.907
28 TIA1	**0.930**	0.924	0.926	0.842	0.896
29 TIAL1	0.893	0.888	**0.895**	0.831	0.858
30 U2AF2	**0.953**	0.941	0.945	0.861	0.873
31 U2AF2(KD)	**0.931**	0.923	0.930	0.840	0.883

DeepBind, DeeperBind, Oli and GraphProt perform on the same datasets with iDeepS. The boldface indicates this performance is the best among the compared methods

**Fig. 2:**
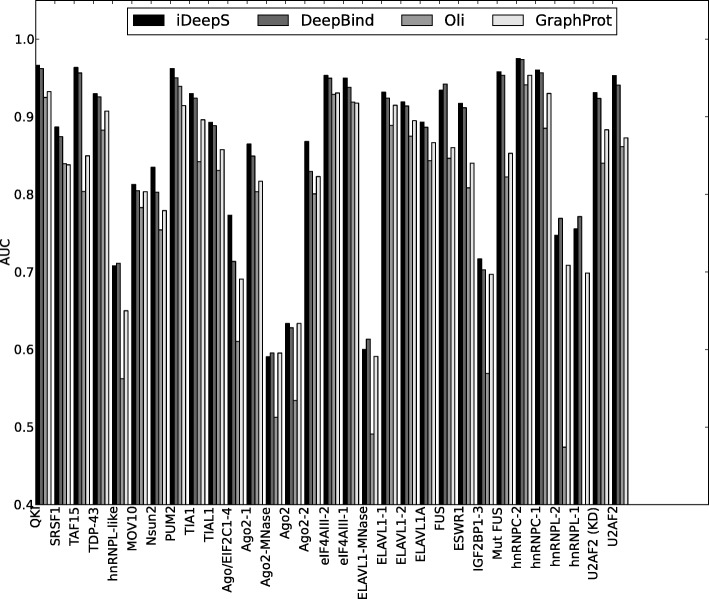
The AUCs of iDeepS, DeepBind, Oli and GraphProt across 31 experiments. The performances are evaluated on the same training and independent testing set across 31 experiments (x-axis) for iDeepS,DeepBind, DeeperBind, Oli and GraphProt. For Oli, DeepBind and DeeperBind, only sequences are used. For iDeepS and GraphProt, sequences and predicted structures are used

Second, we compare iDeepS with structure-profile-based GraphProt, which demonstrates better performance than RNAcontext [7]. Across the 31 experiments, GraphProt yields the average AUC of 0.82, which is worse than 0.86 of iDeepS. As shown in Fig. 2, iDeepS achieves better AUCs than GraphProt on 30 of the 31 experiments. Our method improves the AUCs for some proteins by a large margin. For example, iDeepS yields an AUC 0.77 for protein Ago/EIF, which is an increase of 12% compared to AUC 0.69 of GraphProt (Table 2).

In addition, iDeepS outperforms iONMF (reported average AUC of 0.85 on the same data) using multiple sources of data, including kmer frequency, secondary structure, GO Information and gene type [14]. They also report that the iONMF surpasses the GraphProt and RNAcontext. However, iDeepS performs a little worse than our other deep learning based method iDeep, which integrates multiple sources of data, including gene type and clip-cobinding, instead of only sequences. It is expected that the fully sequence-based method iDeepS will have a more general application scope in the real-world applications.

In summary, iDeepS not only on average achieves better performance than other peer sequence-based methods, it also outperforms some approaches integrating multiple sources of hand-designed features. Our results demonstrate that iDeepS benefits strongly from learning the combination of sequence and structure features for predicting RBP binding sites.

### Insights in sequence-structure motifs

A big advantage of iDeepS is that it also provides biological insights, e.g. learned binding motifs, of the RBPs. As compared to GraphProt, which requires a complicated postprocessing step, iDeepS easily converts learned parameters of the convolved filters to PWMs and allows for identification of the sequence and structure motifs.

**Fig. 3:**
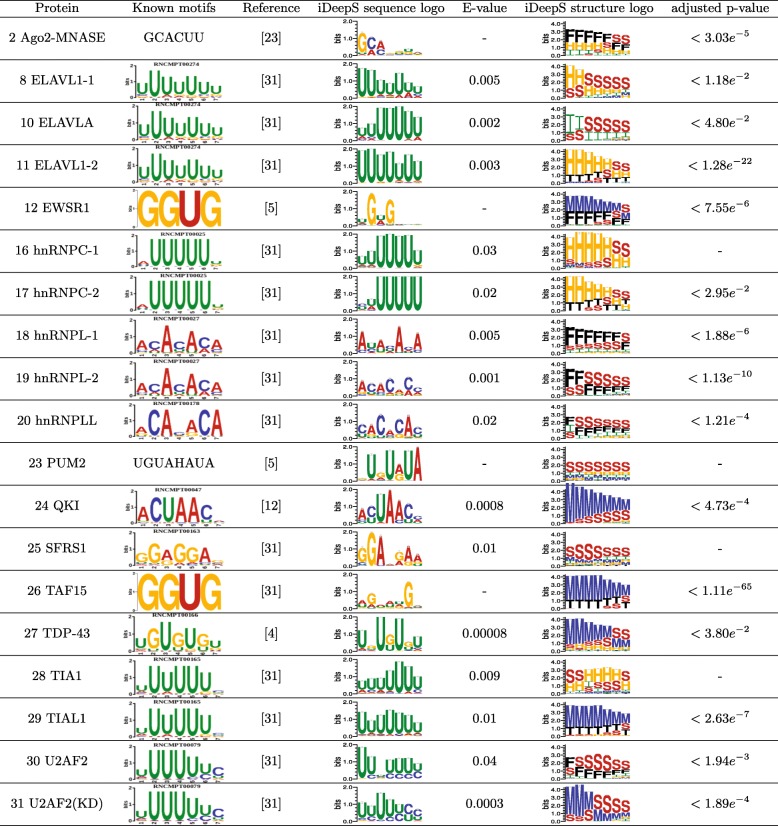
iDeepS captures known sequence motifs and structure motifs. The predicted sequence motifs are compared them against known motifs in study [48] from CISBP-RNA database and literature. E-value is the expected number of false positives for the predicted motifs against known motifs using TOMTOM. The Adjusted p-value is estimated for the corresponding structure motif using enrichment analysis tool AME in MEME Suite. The structure motifs are labelled as follows: stems (S), multiloops (M), hairpins (H), internal loops (I), dangling end (T) and dangling start (F). Note that these listed logos do not represent the full extent of the matched motifs

In this study, we infer the binding motifs across 31 experiments. Of these, 19 experiments have known sequence motifs in the CISBP-RNA database or the literature. As shown in Fig. 3, iDeepS is able to discover experimentally verified sequence motifs for these 19 experiments, of which 15 are matched against CISBP-RNA with significant E-value cutoff 0.05 provided by TOMTOM [33]. The motifs of the remaining 4 proteins resemble the motifs reported by other studies based on visual inspection. iDeepS discovers repeated UG dinucleotides motifs for TDP-43, which contains these dinucleotide repeats in 80% of the 3’UTR region by microarray analysis [13, 34]. iDeepS captures a known motif, which is a crucial regulator in germline development [35], for QKI with significant E-value 0.00008. The motif for PUM2 has been found with an AU-rich sequence motif by iDeepS, which is close to the motifs identified based on top sequence read clusters [7]. The results show that the sequence motifs identified by iDeepS are consistent with verified motifs.

The iDeepS method allows for discovery of structure motifs. iDeepS has demonstrated that RBPs have preferences to generally structured regions. As shown in Fig. 3, the proteins in the ELAVL protein family prefer binding to stem structures, which is consistent with the in vivo and in vitro binding data [36]. iDeepS also discovers that the protein hnRNPC prefers to bind to U-rich hairpin structures, the protein PUM2 binds to stem regions which are UA-rich and the protein QKI interacts with the multiloops region, which all agree with the finding in [13]. Of the 19 structure motifs listed in Fig. 3 that are similar to detected structure motifs by GraphProt, 15 are significantly enriched with adjusted p-value < 0.05 estimated by AME [37].

**Fig. 4:**
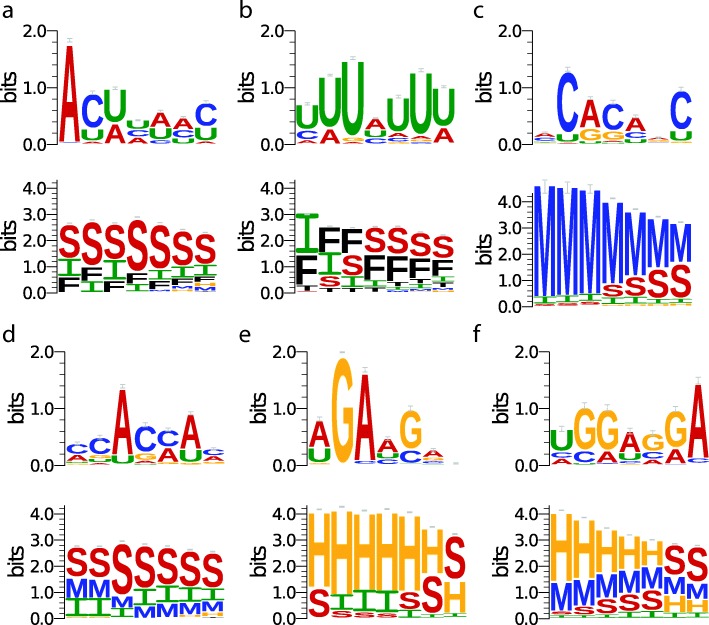
The identified novel binding sequence and structure motifs by iDeepS for RBPs. **a** protein FUS. **b** protein MOV10. **c** protein IGF2BP1-3. **d** protein Ago2. **e** protein EIF4A3. **f** protein NSUN2. In the structure motif logos, they are labelled as follows: stems (S), multiloops (M), hairpins (H), internal loops (I), dangling end (T) and dangling start (F)

We further investigate the identified motifs for FUS, MOV10 and IGF2BP1-3 (Fig. 4), who have no sequence motifs in CISBP-RNA database. FUS has been found to bind to AU-rich stem structure (adjusted p-value: 1.55*e*^−2^ for structure motif) according to study [5], which is captured by iDeepS (Fig. 4a). In addition, we find similar motifs to GraphProt for protein MOV10 with AU rich stem region (Fig. 4b, adjusted p-value: 3.89*e*^−3^ for structure motif), and IGF2BP1-3 protein with CA dinucleotides multiloop region (Fig. 4c, adjusted *p*−value: 5.01*e*^−5^ for structure motif). iDeepS discovers another AC-rich stem-loop motif identified in [38] for Ago2 (Fig. 4d, adjusted *p*−value: 4.28*e*^−2^ for structure motif), which is different from the motif of Ago2 listed in Fig. 3. Compared to GraphProt, iDeepS is able to discover multiple binding sequence and structure motifs for each protein.

We also discover many novel motifs that we could not verify against currently available knowledge. All sequence and structure motifs discovered by iDeep and the reports of their enrichment analysis are available at https://github.com/xypan1232/iDeepS/tree/master/motif. For instance, iDeepS captures novel motifs for RBP EIF4A3 and NSUN2 (Fig. 4e and f), their sequence motifs are enriched with adjusted p-value 5.18*e*^−53^ and 1.53*e*^−8^, respectively. Similarly, their structure motifs are enriched with adjusted *p*−value 4.20*e*^−3^ and 7.02*e*^−5^, respectively. They both show preference for a hairpin region. These discoveries have not been found by any earlier studies and need further verification.

### Added value of BLSTM

**Fig. 5:**
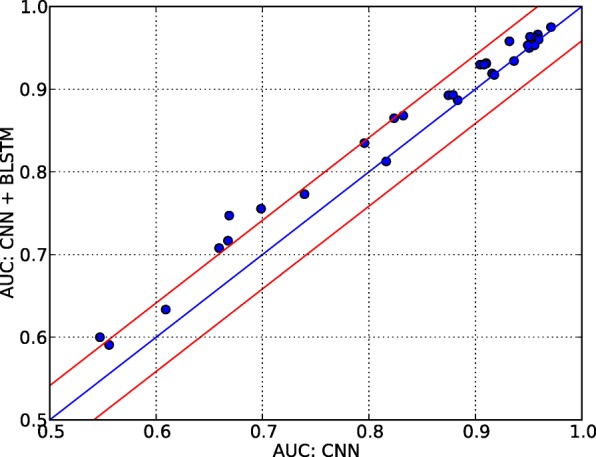
The difference of predictive performance using CNN + BLSTM and only CNN. On the y-axis the performance of the full model with CNNs and BLSTM is shown. The x-axis shows the performance of the model using only the CNNs without BLSTM. The two red lines indicate the 2 times standard deviation of the difference between only using CNN and using CNN + BLSTM

To provide more insights in the added value of the BLSTM we compare the results with a variant using only CNNs and no BLSTM layer. As shown in Fig. 5, iDeepS yields better performance than the variant using only CNNs for most of the 31 experiments. After taking 2 times standard deviation of differences into consideration, iDeepS significantly outperforms the variant only using CNNs on 6 experiments. For the CNN, we optimized the hyper-parameters learning rate and weight decay by a few trials of human-guided search (Additional file 3: Table S1). As shown in Table S1, the performance of the variant is still worse than iDeepS among those tested parameters. Especially a large learning rate of 0.01 will cause the model not to converge, and the performance for some RBPs is similar to random guessing. Based on these results, we decided to use a default learning rate of 0.001 for this study. The results indicate that BLSTM is better able to capture motifs for predicting RBP binding sites, which suggests long-term dependencies between sequences and structures. In addition, iDeepS performs significantly better on 3 experiments than the variant with CNN + BLSTM using only the sequences (Additional file 4: Figure S3), which demonstrates that introducing structure information improves RBP binding site prediction.

DeepBind achieves an average AUC of 0.85 across the 31 experiments by only using sequence CNN, which is a little better than 0.84 of simply concatenating the outputs from sequence and structure CNNs. The reason is that the structure information is predicted from sequences, there exsits correlation between sequences and structures, leading to redundant information, which might hurt the model training. DeepBind performs worse than iDeepS (AUC: 0.86) with the added BLSTM layer after sequence and structure CNNs. The results suggest BLSTM can learn long-term dependencies between sequence and structure motifs, which may reduce the impact of redundant information.

## Discussion

iDeepS is a fully sequence-based method, which will have a more general application scope in the real-world applications than iDeep based on multiple sources of hand-designed features. In addition, the other contribution of iDeepS is to identify the binding sequence and structure of RBPs simultaneously. The iDeepS method has many possible applications. When there are RNA sequences available with potential target sites for RBPs of interest, then these sequences can be fed into iDeepS models. The iDeepS method estimates the probability of those RNA sequences bound to certain RBPs. Pei et al. [39] analyze HT-SELEX data to identify structure motifs for ribosomal protein S15. iDeepS can directly identify the binding sequence and structure motifs of RBPs from sequences. The captured sequence and structure context are an important basis for further research, which could have high clinical impact. For example, these findings could contribute to discovering the mechanisms of diseases involving RBPs. Some structure specificities increase the possibility of the disruption of the structures within binding sites, which might cause diseases, e.g. protein FMR1 in fragile X syndrome [11]. Furthermore, iDeepS has the potential application on predicting the effects of mutations [19, 40]. For example, we can mutate the nucleotides of binding sites, then use iDeepS to predict whether the new binding sites have a big shift compared to experimentally verified sites. In addition, iDeepS can be first used to discover those RBPs that interact with miRNAs, then depletion of those identified RBPs is used to control miRNA expression level [6]. This function is especially of interest for those oncogenic miRNAs in therapeutic applications.

In spite of the promising performance of iDeepS, there still exists some limitations. 1) iDeepS applied the same stringent criteria as described in iONMF [14] to create the negative sites, those negative sites were constructed from genes that were not identified as interaction targets in any of 31 RBPs. This is a strong assumption, which could impact the prediction quality. 2) iDeepS also fails in those RBPs where other existing tools also have low AUC values. The reason might be that the quality of training dataset for those RBPs is low, e.g. high false positives. Thus, more studies are needed to further improve the data quality. 3) Different RBP families show RNA-binding specificities, thus we train a RBP-specific model, a model per RBP. In total, we train 31 models for the 31 experiments in this study, thus iDeepS is only able to predict binding targets for those specific RBPs among these 31 experiments. However, many computational methods [20, 41] train a mixed model with RNA and protein sequences as inputs, and they can predict the binding potential scores for any pairs of RNAs and proteins.

## Conclusion

In this study, we present a fully automatic deep learning method iDeepS to infer both sequence and structure preferences of RBPs and predict the RBP binding sites from RNA sequences. We evaluate iDeepS on RBP binding sites derived from the CLIP-seq datasets. iDeepS is able to predict the RBP binding sites on RNAs with higher accuracy than the state-of-the-art methods. The BLSTM layer in the iDeepS algorithm ascertains long-term dependencies between sequence and structure motifs, which improves its predictive performance. Importantly, the captured motifs align well with the previously reported binding motifs obtained from CISBP-RNA and literature. Moreover, iDeepS also discovers some novel motifs still not experimentally verified. Compared to existing black-box machine learning algorithms, iDeepS is able to find verified sequence and structure binding motifs, which are expected to provide important clues for understanding the biological functional mechanisms of RNA and its binding protein RBP.

## Methods

We develop the computational approach iDeepS (Fig. 1) to predict the RBP binding sites on RNAs. We apply one-hot encoding for the sequences and secondary structures predicted by RNAshapes [42], and feed these into CNNs and a BLSTM to predict RBP binding sites. Finally, we extract the sequence and structure motifs from the learned convolution filters of the CNNs and evaluate them against known verified motifs.

### Datasets

In this study, we train deep learning models for RBP binding sites derived from CLIP-seq data [14] available at (https://github.com/mstrazar/ionmf), where original data are retrieved from DoRiNA [30] and iCount (http://icount.biolab.si/). This CLIP-seq dataset consists of 19 proteins with 31 experiments, including representative RBPs Ago2, TIA1 and ELAVL1. For each experiment, each nucleotide within clusters of interaction sites derived from CLIP-seq were considered as binding sites. The negative sites were sampled from within genes that were not identified as interaction sites in any of the 31 experiments. In each experiment, a total 24,000 samples are used for training, 6,000 samples for model optimization and validation, and the other 10,000 samples for independent testing, they are used to train and evaluate a RBP-specific model.

### Encoding sequence and structure

The RNA sequence is used as a one-hot representation encoded into a binary matrix, whose columns correspond to the presence of A, C, G, U and N [19, 43]. Given a RNA sequence *s*=(*s*_1_,*s*_2_,...,*s*_*n*_) with n nucleotides and sequence motif detector with defined size m, the binary matrix M for this sequence is represented as follows:

1 $$ M_{i,j}=\left\{ \begin{array}{ll} 0.25 \: if \: s_{i-m+1} = N \: or \: i < m \: or \: i > n- m\\ 1 \: if \: s_{i-m+1} \: is \: (A,C,G,U) \\ 0 \: otherwise \end{array} \right.  $$

where *i* is the index of the nucleotide, *j* is the index of the column corresponding to A, C, G, U.

We use abstract secondary structure annotation from RNAshapes [42] implemented in https://github.com/fabriziocosta/EDeN. The RNAshapes have six generic shapes: stems (S), multiloops (M), hairpins (H), internal loops (I), dangling end (T) and dangling start (F). For each sequence s, we obtain the structure shapes *str*=(*str*_1_,*str*_2_,...,*str*_*n*_) by RNAshapes, which are converted into a binary matrix R with columns corresponding to the presence of F, H, I, M, S, T, and with k representing the predefined structure motif size.

2 $$ R_{i,j}=\left\{ \begin{array}{ll} 0.16 \: if \: i < k \: or \: i > n- k\\ 1 \: if \: {str}_{i-k+1} \: is \: (F, H, I, M, S, T)\\ 0 \: otherwise \end{array} \right.  $$

where *i* is the index of the structure, *j* is the index of the column corresponding to S, M, H, I, T, F.

### Convolutional neural network

The Convolutional Neural Network (CNN) [44] is inspired by the animal visual cortex. It consists of convolution, activation, and max-pool layers.

The one-hot encoding matrix derived from RNA sequences and structures are the inputs to the CNNs and are used to learn the weight parameters of the convolution filters. The convolution layer outputs the matrix inner product between input matrix and filters. After convolution, a rectified linear unit (ReLU) is applied to sparsify the output of the convolution layer and keep only positive matches to avoid the vanishing gradient problem [45]. Finally, a max pooling operation is used to reduce the dimensionality and yield invariance to small sequence shifts by pooling adjacent positions within a small window.

Before feeding into the next layer, the CNNs of sequence and structure are merged into one layer. The subsequent layers of the iDeepS act jointly on the merged sequence and structure layers.

### Long Short Term Memory networks

LSTM belongs to the class of recurrent neural network [46], it incorporates long-term dependent information to assist the present prediction. In this study, LSTM is used to identify informative combinations of the extracted sequence and structure motifs [27], which projects the original input into a weighted representation.

As the LSTM sweeps across each element of the input, it first decides which information should be excluded by a forget gate layer based on previous inputs. Then an input gate layer is used to determine which information should be stored for the next layer, and updates the current state value. Finally, an output gate layer determines what parts of state value should be output. Taking a sequence $\{\mathbf {x}\}_{t=1}^{T}$ as input, the LSTM have the hidden states $\{\mathbf {h}\}_{t=1}^{T}$, cell states $\{\mathbf {C}\}_{t=1}^{T}$, and it outputs a sequence $\{\mathbf {o}\}_{t=1}^{T}$. The above steps can be formulated as follows:

3 $$\begin{array}{*{20}l} \mathbf{f}_{t}&= \sigma\left(\mathbf{W}_{f}\mathbf{x}_{t}+\mathbf{U}_{f}\mathbf{h}_{t-1}+\mathbf{b}_{f}\right), \\ \mathbf{i}_{t}&= \sigma\left(\mathbf{W}_{i}\mathbf{x}_{t}+\mathbf{U}_{i}\mathbf{h}_{t-1}+\mathbf{b}_{i}\right), \\ \mathbf{c}_{t}&= \mathbf{f}_{t}\odot\mathbf{c}_{t-1}+\mathbf{i}_{t}\odot\text{tanh}\left(\mathbf{W}_{c}\mathbf{x}_{t}+\mathbf{U}_{c}\mathbf{h}_{t-1}+\mathbf{b}_{c}\right), \\ \mathbf{o}_{t}&= \sigma\left(\mathbf{W}_{o}\mathbf{x}_{t}+\mathbf{U}_{o}\mathbf{h}_{t-1}+\mathbf{b}_{o}\right), \\ \mathbf{h}_{t}&=\mathbf{o}_{t}\odot\text{tanh}(\mathbf{c}_{t}) \end{array} $$

where ⊙ denotes element-wise multiplication, the *σ* is the Logistic Sigmod function and *tanh* is the tanh function to force the values to be between -1 and 1. **W**_*f*_, **W**_*i*_, **W**_*o*_, **U**_*f*_, **U**_*i*_ and **U**_*o*_ are the weights and **b**_*f*_, **b**_*i*_, **b**_*c*_ and **b**_*o*_ are the bias.

In iDeepS, a bidirectional LSTM (BLSTM) is used, i.e., it sweeps from both left to right and right to left, and the outputs of individual directions are concatenated for subsequent classification.

### Identifying the binding sequence and structure motifs

To explore the learned motifs, we investigate the learned filters of sequence and structure CNNs in iDeepS. We convert them into position weight matrices (PWM) like DeepBind and Basset [19, 40], which are matched against input sequences and structures to discover binding motifs.

Assuming we have a sequence or structure *S*_*m*_ and a convolve filter with size L, if the activation value *A*_*mfi*_ of filter f at position i is greater than 0.5 max*miA*_*mfi*_, then this sequence or structure in windows L centring the position i is selected to align sequence motifs using WebLogo [47].

4 $$ A_{mfi} = ReLU\left(\sum_{l=1}{\sum\limits_{d=1}^{D}{w_{fld}*s_{m, i+1,d}}}\right)  $$

where *ReLU*(*x*)=*max*(0,*x*), *w*_*f*_ is the weights of filter *f*, *m* is the sequence length. For sequence motifs, D is 4. For structure motifs, D is 6.

To verify the predicted sequence motifs, we align them against 102 known motifs in study [48] from CISBP-RNA using the TOMTOM algorithm [33] with *p*−value < 0.05. For some proteins, currently there are still no verified motifs in the CISBP-RNA database, we investigate them via the literature.

Furthermore, we also calculate motif enrichment scores of predicted sequence and structure motifs using AME [37] in the MEME suite [8]. Fisher’s exact test is used to estimate the *p*−values, which are adjusted for multiple tests using a Bonferroni correction. Take sequence motifs as an example, we first scan the predicted motifs against the input sequences, and do the same for the shuffled sequences considered as the background sequences. Then we compare them to calculate the enrichment scores. We do the same enrichment analysis for predicted structure motifs.

### Implementation

The iDeepS is implemented in python using keras 1.1.2 library https://github.com/fchollet/keras. We set the maximum number of epochs to 30, and the batch size to 50. The validation dataset is used to monitor the convergence during each epoch of the training process, so the training process can be stopped early. The model is trained by back-propagation using categorical cross-entropy loss, which is minimized by RMSprop [49]. In addition, we employ multiple techniques to prevent or reduce over-fitting, e.g. batch normalization [50], dropout [51] and early stopping.

The number of motifs for both sequence and structure CNNs is set to 16 as suggested by DeepBind [19]. As indicated in iDeep [28], ReLU leads to information loss for some bits in motifs. As proposed by DeepBind, the the filter_length (motif width) should be 1.5 times the verified motif width, which is 7 in CISBP-RNA database [48]. Therefore, we choose a filter length of 10 in this study. When converting the filters to PWMs, we only use the first 7 bits of 10.

### Baseline methods

There are many computational methods developed for predicting RNA-protein binding sites [13, 14, 19, 31]. In this study, we compare iDeepS with the state-of-the-art sequence-based methods DeepBind [19], DeeperBind [23], Oli [31], iONMF [14] and GraphProt [13]. DeepBind, uses a sequence CNN with the same architecture as iDeepS to predict RBP binding sites. For GraphProt (v1.1.3), it encodes the sequence and structure into high-dimensional graph features, which are fed into a SVC to classify RBP bound and unbound sites. In this study, we use a window size of 80 in GraphProt and the other parameters are set to the default. iONMF uses matrix factorization to predict RBP binding sites by integrating different sources of features [14]. Oli uses linear SVC to classify RBP binding sites based on tetranucleotide frequency features [31]. The performance is measured using the area under the receiver operating characteristic (ROC) curve (AUC).

## Additional files

Additional file 1 **Figure S1**. The network architectures of iDeepS. (PNG 45 kb)

Additional file 2 **Figure S2**. The AUCs of using DBN and k-mer features to predict RBP binding sites. (EPS 54.4 KB)

Additional file 3 **Table S1**. The AUCs of using CNNs with sequence and structure information for different hyperparameters learning rate and weight decay. (PDF 47 kb)

Additional file 4 **Figure S3**. The difference of predictive performance using sequence + structure and only sequence. On the y-axis the performance of the full model with sequence + structure is shown. The x-axis shows the performance of the model using only sequences. The two red lines indicate the 2 times standard deviation of the difference between only using sequence and using sequence + structure. (EPS 39 kb)

## Abbreviations

AUC: Area under the ROC curve
BLSTM: Bidirectional long short term memory network
CNN: Convolutional neural network
DBN: Deep belief network
FCL: Fully connected layer
PWM: Position weight matrix
RBPs: RNA binding proteins
ROC: Receiver operating characteristic
