# Supplementary figures and images for: Prediction of RNA-protein sequence and structure binding preferences using deep convolutional and recurrent neural networks
Source: BMC Genomics. 2018 Jul 3;19:511. doi: 10.1186/s12864-018-4889-1 (PMC6029131; doi:10.1186/s12864-018-4889-1)

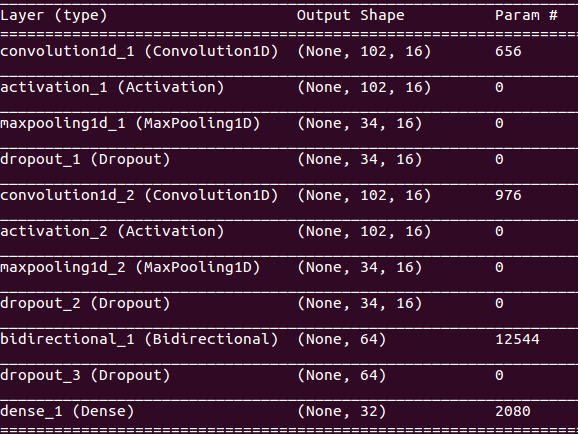

Supplement: Supplementary file 1 — Figure S1. The network architectures of iDeepS. (PNG 45 kb) [file 12864_2018_4889_MOESM1_ESM.png]
