# Supplementary material for: Prediction of RNA-protein sequence and structure binding preferences using deep convolutional and recurrent neural networks
Source: BMC Genomics. 2018 Jul 3;19:511. doi: 10.1186/s12864-018-4889-1 (PMC6029131; doi:10.1186/s12864-018-4889-1)
Supplement: Supplementary file 3 — Table S1. The AUCs of using CNNs with sequence and structure information for different hyperparameters learning rate and weight decay. (PDF 47 kb) [file 12864_2018_4889_MOESM3_ESM.pdf]

Table S1: **The AUCs of using only CNNs with sequence and strcutre information for different hyperparameters learning rate (lr) and weight decay (wd).**

Protein	lr=0.001, wd=0.001	lr=0.001, wd=0.0001	lr=0.01, wd=0.0001	lr=0.001, wd=0.01	lr=0.0001, wd=0.0001
1 Ago/EIF	0.742	0.741	0.5	0.673	0.670
2 Ago2-MNase	0.591	0.595	0.5	0.555	0.563
3 Ago2-1	0.835	0.847	0.5	0.750	0.765
4 Ago2-2	0.846	0.849	0.815	0.767	0.764
5 Ago2	0.632	0.630	0.587	0.629	0.628
6 eIF4AIII-1	0.943	0.938	0.5	0.908	0.913
7 eIF4AIII-2	0.948	0.953	0.940	0.924	0.925
8 ELAVL1-1	0.926	0.926	0.5	0.915	0.918
9 ELAVL1-MNase	0.603	0.604	0.5	0.568	0.570
10 ELAVL1A	0.886	0.890	0.5	0.873	0.872
11 ELAVL1-2	0.915	0.916	0.907	0.900	0.902
12 ESWR1	0.915	0.916	0.5	0.887	0.889
13 FUS	0.941	0.942	0.935	0.915	0.914
14 Mut FUS	0.953	0.955	0.5	0.913	0.924
15 IGFBP1-3	0.711	0.712	0.5	0.668	0.673
16 hnRNPC-1	0.958	0.958	0.5	0.951	0.951
17 hnRNPC-2	0.975	0.975	0.5	0.970	0.969
18 hnRNPL-1	0.782	0.782	0.767	0.767	0.769
19 hnRNPL-2	0.779	0.782	0.5	0.765	0.762
20 hnRNPL-like	0.732	0.731	0.690	0.714	0.713
21 MOV10	0.805	0.806	0.796	0.793	0.796
22 Nsun2	0.861	0.862	0.842	0.834	0.828
23 PUM2	0.946	0.956	0.5	0.922	0.920
24 QKI	0.962	0.963	0.5	0.946	0.949
25 SRSF1	0.872	0.870	0.5	0.828	0.838
26 TAF15	0.956	0.959	0.946	0.931	0.937
27 TDP-43	0.927	0.927	0.5	0.917	0.919
28 TIA1	0.926	0.927	0.916	0.919	0.919
29 TIAL1	0.884	0.885	0.5	0.876	0.875
30 U2AF2	0.941	0.945	0.5	0.930	0.933
31 U2AF2(KD)	0.923	0.926	0.5	0.912	0.912
